# Design, Development and Validation of a Knee Brace to Standardize the US Imaging Evaluation of Knee Osteoarthritis

**DOI:** 10.1109/JTEHM.2021.3137628

**Published:** 2021-12-22

**Authors:** A. Sorriento, A. Cafarelli, P. Spinnato, A. Russo, G. Lisignoli, F. Rabusseau, P. Cabras, E. Dumont, L. Ricotti

**Affiliations:** BioRobotics Institute, Scuola Superiore Sant’Anna 56127 Pisa Italy; Department of Excellence in Robotics and AIScuola Superiore Sant’Anna 56127 Pisa Italy; Diagnostic and Interventional RadiologyIRCCS Istituto Ortopedico Rizzoli 40136 Bologna Italy; Clinica 2, IRCCS Istituto Ortopedico Rizzoli 40136 Bologna Italy; SC Laboratorio di Immunoreumatologia e Rigenerazione TissutaleIRCSS Istituto Ortopedico Rizzoli 40136 Bologna Italy; Image Guided Therapy (IGT) 33600 Pessac France

**Keywords:** Acquisition standardization, osteoarthritis, tele-ultrasonography, ultrasound imaging

## Abstract

Objective: A repeatable and reliable follow-up of knee injuries would be desirable to prevent delayed diagnosis and to monitor the efficacy of the applied treatment over time. Ultrasound (US) techniques are an attractive option to this purpose, since they are safe, low-cost and non-invasive. However, its use in the clinical practice is limited by the high dependency on the operator’s experience. Hence, the objective of this study is to provide a standardization of the US image acquisition process for knee osteoarthritis (OA) allowing an extended clinical use of US technologies in this domain. Methods: Clinical specifications were provided by expert musculoskeletal radiologists thus identifying the subject poses and the US probe positions needed to evaluate the cartilage structure, signs of synovitis and joint effusion. Such considerations were used to derive the technical requirements needed for the development of a wearable brace equipped with specific openings to guide the correct placement of the probe. The feasibility of the developed wearable brace was tested on three healthy volunteers, which were asked to acquire informative US images, similar to the reference images performed by the musculoskeletal radiologist. Results: Thanks to the knee brace, the untrained subjects were able to self-acquire informative B-mode images comparable to the corresponding images acquired by an expert clinician. Discussion/Conclusion: The use of a knee brace intended for knee OA US diagnosis demonstrated the possibility to standardize the acquisition protocol and make its application achievable also for untrained subjects, representing a key step toward tele-ultrasonography.

## I. Introduction

Osteoarthritis (OA) is a degenerative joint disease that mainly affects cartilage, subchondral bone and synovial tissues [1]. OA causes severe pain and joint motion limitations, significantly reducing the quality of life of a large fraction of the population [2]. Despite the significant improvements achieved by the introduction of modern diagnostic techniques, existing imaging methods have still limitations in daily clinical practice. Although conventional radiography is widely used for the assessment of knee OA due to its simplicity and accessibility, it is associated with significant weaknesses, such as the use of ionizing radiations and the inability to directly visualize the articular cartilage or menisci [3]. Magnetic resonance imaging (MRI) is considered the most accurate imaging technique for OA diagnosis [4]; indeed, several structures that are relevant for the functional integrity of the joint, including the cartilage, can be accurately visualized. However, despite its high sensitivity, MRI is not routinely used for the assessment of knee OA because time consuming and expensive [4]. Hence, alternative diagnostic methods for OA assessment, possibly reliable and easily accessible, are highly desirable.

Conventional ultrasound (US) B-mode images are widely used in radiology for the diagnosis of several soft tissues [5]. US techniques offer the advantage to be non-invasive, safe, fast, portable, and low cost. However, they are featured by an intrinsic lower image contrast with respect to MRI. Although US currently plays only a minor role in OA knee assessment clinical routine, its potential has been recently demonstrated. Saarakkala *et al.* [6], explored the diagnostic performance of US imaging for the detection of degenerative changes of the articular cartilage. The authors reported a good agreement of US indications with the Noyes’ arthroscopic grading scale used as the gold standard. Podlipska *et al.* [4], compared US imaging and conventional radiography for the diagnosis of knee OA, using MRI as a standard reference. Results showed that US imaging allows higher accuracy than traditional conventional radiography in the detection of osteophytes in the medial compartment of the knee joint. On the other hand, in the lateral compartment the performance of US was slightly lower than conventional radiography with respect to MRI results.

Despite such interesting results, US is still affected by some issues that hamper its extensive use in the clinical practice. US imaging is a quite strongly operator- and system-dependent diagnostic modality [7]: indeed, the results can be affected by the position and inclination of the probe [8], the contact force between the probe and skin, the acoustic parameters setting (*i.e.*, focus, depth, gain and transmission frequency) and the experience of the operator. All these aspects result in a rather high variability and interpretational doubts on the results obtained by different operators [9], often with the necessity of additional diagnostic confirmations. To overcome the above-mentioned limitations, new methods to standardize US-based diagnostic procedures are needed.

This study proposes a novel standardized echography acquisition modality for knee OA diagnosis. First, standard poses for the patients and specific positions for the US probe were identified based on the know-how of experienced clinicians in the field and driven by considerations available in the scientific literature [6], [10]. Such clinical indications were translated into technical specifications for the development of a wearable brace that could act as a guide for a standardized US image acquisition protocol. The wearable brace was provided with some openings, which allowed an easy and univocal identification of the probe positioning, thus enabling the acquisition of informative images also from not skilled operators such as the patient him/herself.

A standardized acquisition protocol may pave the way for the use of tele-ultrasonography in OA diagnosis. A remote diagnosis of OA could facilitate frequent evaluations, allowing the early detection of complications and enabling the diagnosis also for people that cannot reach the clinical centers.

## II. Materials and Methods

### A. Clinician’s Indications

**FIGURE 1.**
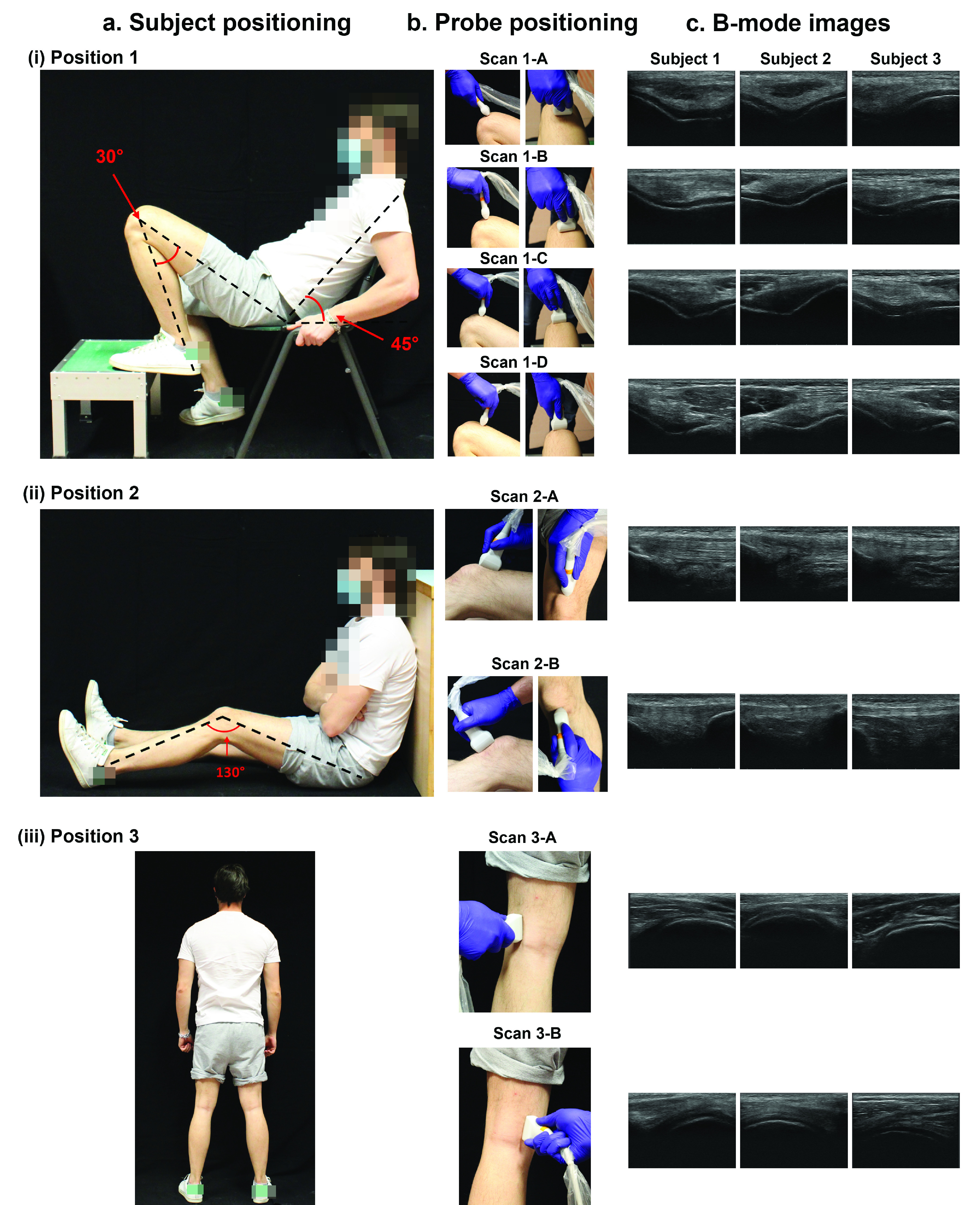
Clinicians’ indications on patient and ultrasound probe positioning for US imaging of the cartilage. In panel a-(i) the subject is seated on a chair in Position 1 and different scanning planes are analyzed: Scan 1-A, Scan 1-B, Scan 1-C and Scan 1-D (b-(i)), to assess the anterior cartilage (c-(i)). In panel a-(ii), the subject is seated on the floor in Position 2 and two scanning planes are analyzed: Scan 2-A and Scan 2-B (b-(ii)), to visualize the synovium and the Hoffa’s adipose body (c-(ii)). In panel a-(iii) the subject is standing in Position 3 and two scanning regions are analyzed: Scan 3-A and Scan 3-B (b-(iii)), to assess the posterior cartilage (c-(iii)).

A musculoskeletal radiologist with 12 years of experience in musculoskeletal imaging provided specific indications for a complete diagnosis of joint diseases, also according to considerations available in literature [6], [10]. Based on these indications, three main poses were identified to be taken by the patients during the procedure for a complete knee US diagnosis. In Position 1 the subject was seated on a chair (height: ~45 cm), with the leg under investigation positioned on a support (height: ~25 cm), as shown in Figure 1(a-(i)). The trunk was flexed at about 45° with respect to the vertical position and the leg was flexed at 30° with respect to the thigh thus to move down the patella, uncovering the cartilage. With this configuration of the subject, it was possible to visualize the axial view of the femoral condyles and trochlear groove placing the US probe above the upper aspect of the patella from different scanning planes (Scan 1-A, Scan 1-B, Scan 1-C, Scan 1-D - Figure 1-b-(i)). The US probe was moved from a starting point (Scan 1-A, right above the patella) until an ending point (Scan 1-D), displaying the anterior cartilage at different levels (Figure 1-c-(i)).

In Position 2 (see Figure 1-a-(ii)), the subject was seated on the floor or a rigid support (e.g., a table), with glutes and heels at the same height, and with the back leaning against a wall or another support. An angle of about 130° between the thigh and the leg was set. In this position, two different imaging planes were identified (Figure 1-b-(ii)) to evaluate the synovium above the quadriceps tendon (Scan 2-A) and the Hoffa’s adipose body above the patellar tendon (Scan 2-B), as shown in Figure 1(c-(ii)). For Position 1 and 2 the correct angles were measured using a goniometer.

Finally, in Position 3 (see Figure 1-a-(iii)) the subject was standing up with the knee completely extended, thus enabling posterior cartilage visualization (Figure 1-c-(iii)). The probe was positioned in the popliteal fossa above lateral (Scan 3-A) and medial femoral condyles (Scan 3-B) of the knee to visualize the posterior cartilage (see Figure 1-b-(iii)).

All US measurements were carried out using an ArtUS EXT-1H system (Telemed, UAB, Lithuania) equipped with a 192 elements linear probe L15-7H40-A5 with a transmission frequency of 15 MHz. The depth was set at 30 mm for the evaluation of the anterior cartilage and 40 mm for the posterior cartilage. The contact area between the probe and the skin was about $1\times $ 5 cm^2^. To check for the repeatability of the acquisition method, three male subjects (age =31 ± 2.3 years, weight = 77 ± 4.6 Kg, height = 181.3 ± 2 cm) were recruited for collecting reference B-mode images with the procedure described above. All such images were acquired by the same clinician.

All subjects gave written informed consent, and the study was conducted in accordance to the institutional ethics guidelines and procedures.

### B. Wearable Brace Design and Development

**FIGURE 2.**
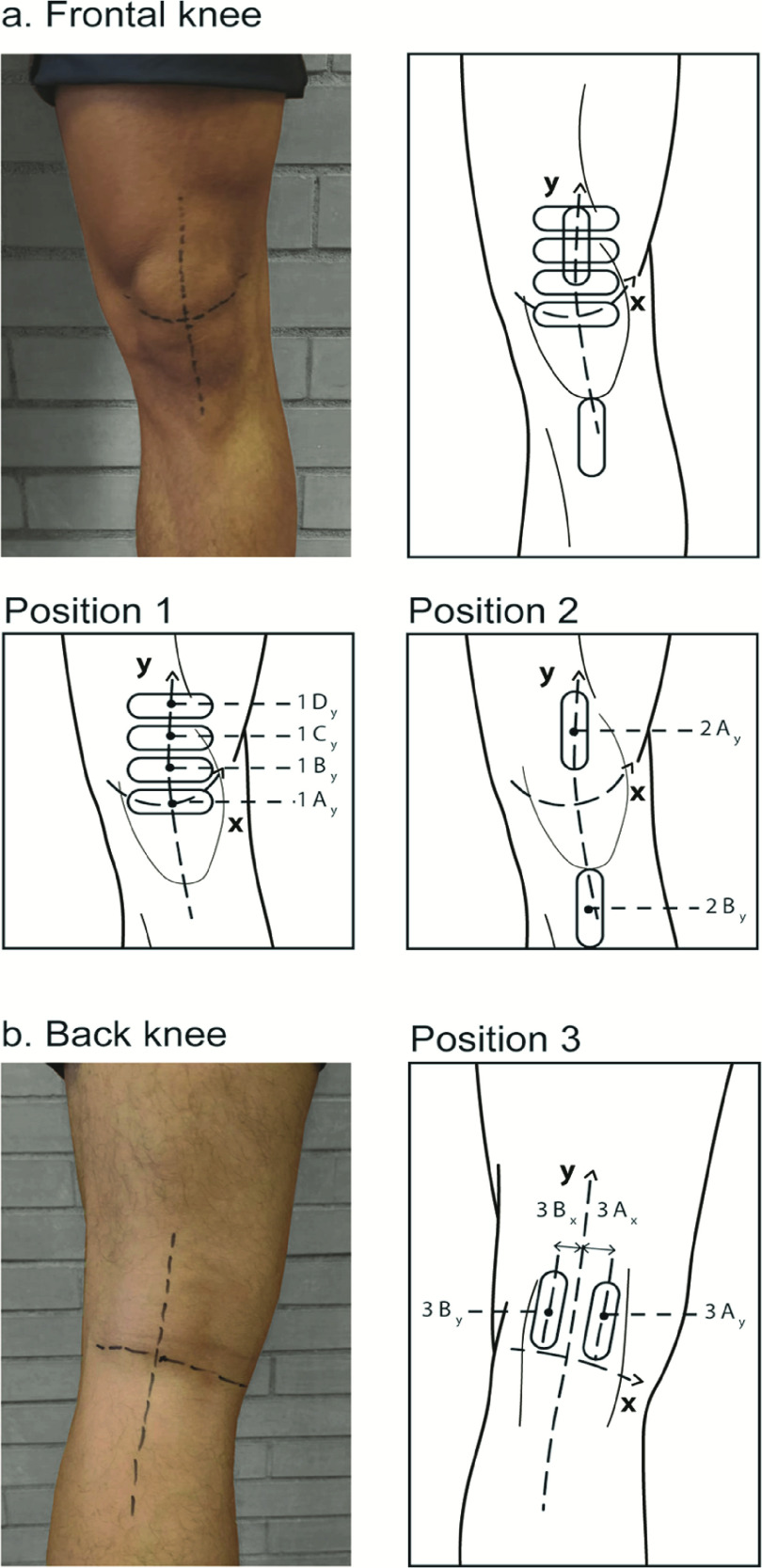
Reference positions for the US probe positioning in the wearable brace design. In panel a, the probe locations with respect to the reference system of the frontal knee are shown, while the subject is in position 1 and 2. Similarly, in panel b, the positions of the probe for the assessment of the posterior cartilage are identified using the reference system of the back knee.

The probe positions (*i.e.*, location of the probe with respect to the reference systems shown in Figure 2) were measured for the three recruited subjects, thus providing the relevant geometric information needed for the brace design process. For Position 1 and Position 2, two orthogonal axes were drawn by considering the centre of the patella (in extended knee position) as the centre of the reference system. As regards Position 3, the natural bending line of the back knee (popliteal fossa) was taken as the x axis, while the orthogonal y axis crossed the centre of the posterior knee. For each scan, the coordinates x and y of the precise locations of the probe centre were measured and the results are reported in Table 1.

**TABLE 1:** Measurements of the x, y Coordinates of the Probe Centers for Each Probe Location. These Values Were Calculated With Respect to the References Systems Drawn on the Knee and Displayed in Table 1

Probe location (centre)	x-coordinate [cm]	y-coordinate [cm]
1A	0 ± 0	0 ± 0
1B	0 ± 0	1.8 ± 0
1C	0 ± 0	3.8 ± 0
1D	0 ± 0	5.3 ± 0.6
2A	0 ± 0	4.2 ± 0.6
2B	0 ± 0	−5.3 ± 1.3
3A	3.7 ± 0.3	2.5 ± 0
3B	−4 ± 0.5	2.5 ± 0

A guidance system for the position of the US probe was developed by using a commercial brace (Dr. Arthritis, Berkshire, UK) in which openings were produced in correspondence to the results reported in Table 1. The openings were provided with silicone inserts specifically designed to house the US probe. Silicon material assures robustness as well as enough flexibility even when the knee is totally bent (see position 1, Figure 2, a-(i)). Two molds were designed (one for the frontal inserts and the other one for the back inserts) and 3D printed using a Creality Ender 3 printer (Creality, China). The silicon (RTV 145C, Résines & Moulages, France) was poured inside such molds and left to cure for 24 hours. Then, the silicon inserts were glued in the brace openings, which were previously cut, using Loctite 4062 glue for the anterior brace and Pattex Contact Neoprene glue for the posterior brace. A lining layer was used to cover the inserts of the posterior brace, avoiding friction between skin and silicone thus simplifying the brace wearing.

The silicone inserts were designed according to both the dimension of the probe and the variability of the subjects (see Table 1) trying to reduce to a minimum the displacement of the probe inside the opening so as to increase the positioning precision. Furthermore, the raised wall design of the inserts helps maintaining the probe perpendicular to the skin (as required in the diagnostic examination).

### C. Self-Acquisition of Us Images

To test the usability of the developed wearable knee brace, the same three subjects involved in the US measurements described above were asked to wear the brace and to perform a US self-acquisition, trying to reproduce the corresponding reference clinical images (Figure 1-c) displayed on a screen. Since the subjects had difficulties in executing a self-acquisition in the standing position (i.e., Position 3–Figure 1-a-(iii)), the scanning of the posterior cartilage was performed with the subject seated and the leg completely extended (Figure 4-a-(iii) in the Results section).

**FIGURE 3.**
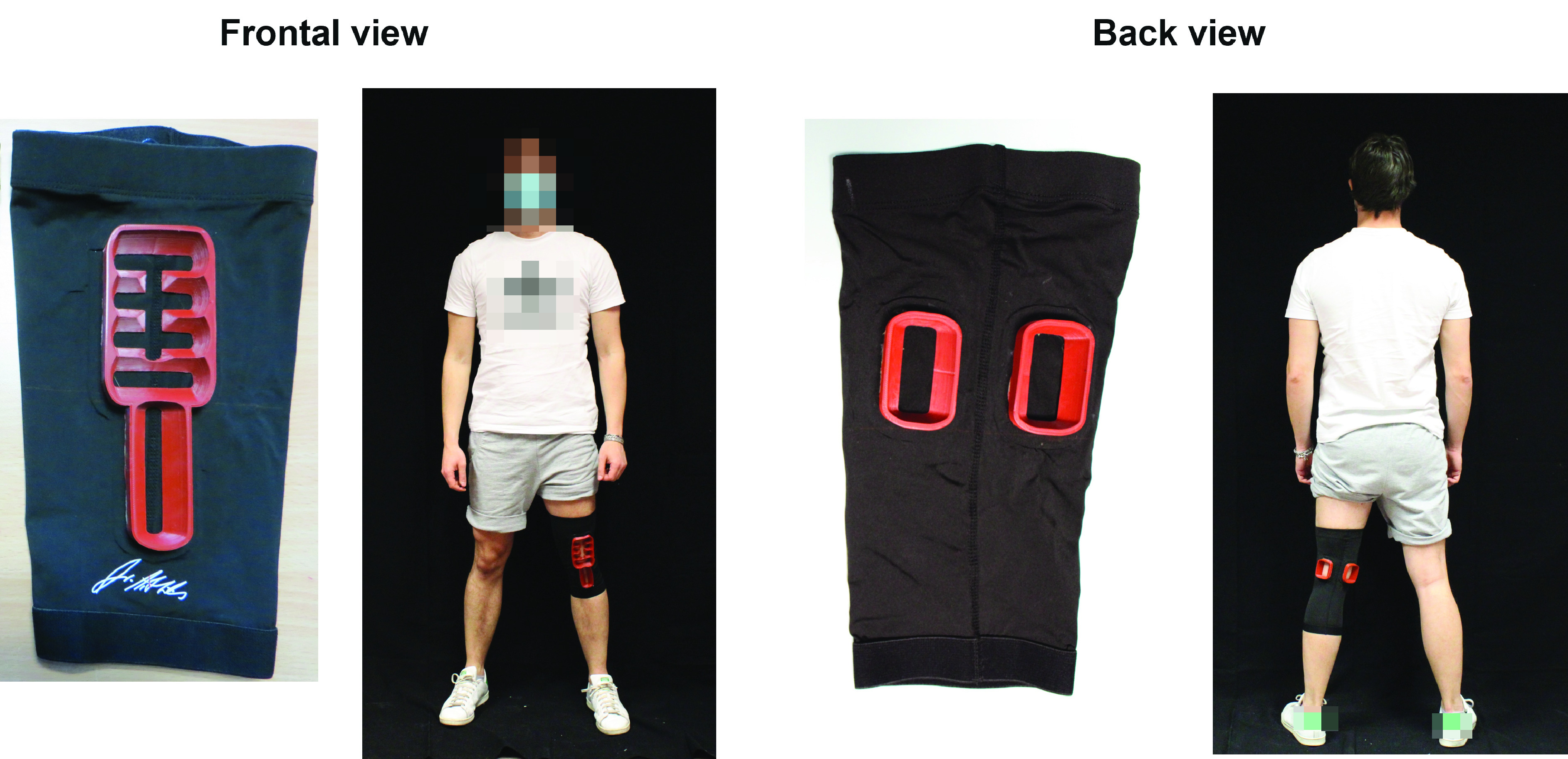
Frontal and back views of the brace and a subject wearing it. In panel a, the frontal side of the brace is shown, in which the anterior openings can be observed (i) for the US probe positioning. A subject wearing the system in the frontal view is shown in (ii). In panel b, the back side of the brace is shown, in which the back openings can be observed (iii) for the US probe positioning. A subject wearing the system in the back view is shown in (iv).

**FIGURE 4.**
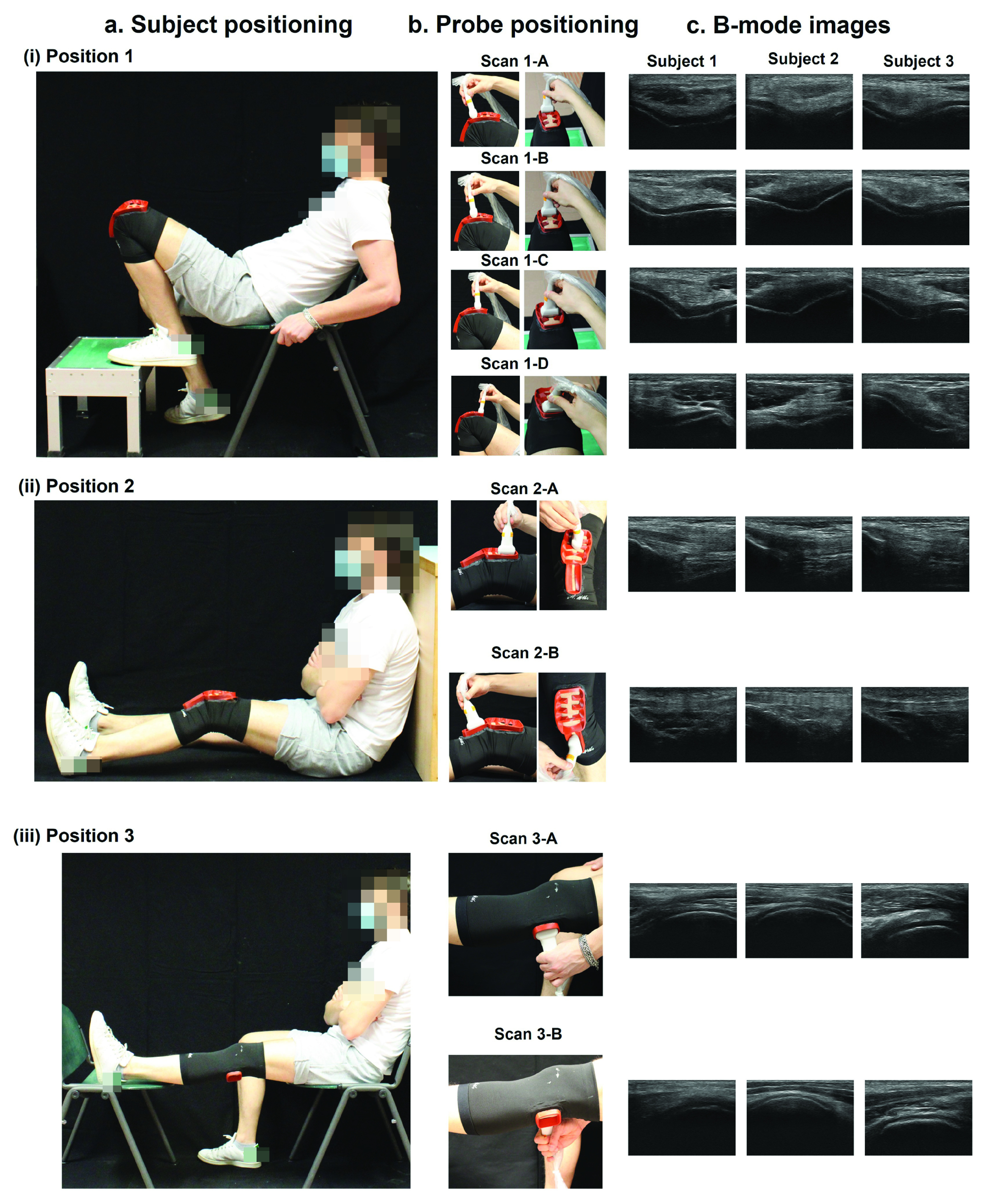
Results of the brace testing. In panel a-(i), the subject is seated on a chair in Position 1 to perform a self-acquisition of the anterior cartilage (c-(i)) by using the openings of the wearable brace (b-(i)). In panel a-(ii), the subject is seated on the floor in position 2 to visualize the synovium and the Hoffa’s adipose body (c-(ii)) with the support of the corresponding brace openings (b-(ii)). In panel a-(iii), the subject is seated on a chair with the knee completely extended to acquire US images of the posterior cartilage (c-(iii)) by using the back inserts of the brace (b-(iii)).

## III. Results

### A. Wearable Brace Design and Development

In Table 1, the x and y coordinates of the probe location are reported for each scanning position with respect to the reference systems shown in Figure 2. For each location, data are reported as mean ± standard deviations among the measurements made on the three subjects.

Based on these data, specific openings were created on the anterior and posterior parts of the brace (see Figure 3). Figure 3 also shows a subject wearing the brace (frontal and back views).

### B. Self-Acquisition of Us Images

The three subjects wore the brace without any problem and were able to reproduce the three positions identified as the optimal ones to image the cartilage (Figure 4a), as indicated in the “Clinicians’ indications” section. Then, for each position and each opening of the brace (Figure 4b), the subjects performed a US self-acquisition, trying to reproduce the corresponding images acquired by the clinician.

The B-mode images acquired by the three subjects, shown in Figure 4c, resulted very similar to the ones acquired by the clinician without the use of the brace Figure 1c), demonstrating the ability of the brace to guide non-expert users in taking images having a satisfactory quality for diagnosis. The acquisitions in the Position 2 were more difficult to conduct compared to Positions 1 and 3 and thus the subjects needed more time to get the correct images. A more detailed comparison between the images acquired by the subjects and the ones originally taken by the clinician is shown in Figures S1-S8.

## IV. Discussion

US-based knee joint evaluation has advantages over other diagnostic techniques. Indeed, it allows direct, safe and low-cost monitoring of cartilage tissue status and other relevant changes such as the presence of osteophytes, inflammation, synovial thickening, joint effusion, tendons and ligaments and to derive unique information even in the early stage of diseases such as OA [11], [12]. However, conventional US techniques suffer from low reproducibility due to their high dependency on the examiner experience. In addition, the inaccurate visualization of the subchondral bone and the lack of objectivity restrict the use of US imaging in the clinical setting as a standalone modality: it is actually mainly employed in addition to conventional radiography and MRI [3]. The standardization of US image acquisition may have a radical impact on the diagnosis of OA, leading to a universal-acceptable, non-invasive, low-cost and repeatable method among clinicians for pathology assessment and evolution. In this study we proposed a standardized method for the acquisition of US images of the knee joint, aiming to reduce the operator-dependent variables in the diagnosis of OA.

In this regard, a wearable brace enabling a guided US imaging acquisition procedure has been designed and developed starting from clinical indications provided by an expert clinician in the field. The clinical guidelines led to identify three poses of the subject in which the cartilage tissue and the possible inflammatory joint status could be evaluated, and OA-like changes identified (see Figure 1). For example, in Position 1, changes in the morphology of the anterior cartilage could be detected, while in Position 2 inflammations of the Hoffa’s adipose body or joint effusions could be identified. Finally, in Position 3, the posterior cartilage of femoral condyles could be analyzed.

Since the posterior silicon inserts may represent an obstacle when the subject had to bend the knee in Position 1, two versions of the brace were produced: the former enabling the acquisition of the anterior cartilage with the knee bent in Position 1 and 2 (see Figure 3, panel a), the latter enabling the acquisition of the posterior cartilage with the extended leg in Position 3 (see Figure 3, panel b). The brace was tested on the same three healthy subjects employed for deriving the clinical indications. They were asked to wear the knee brace and to perform a self-acquisition guided by the brace openings trying to match the corresponding reference US images, provided by the clinician. Before starting the acquisition, a training session of ~10 min was performed, in which the clinician instructed the subject on the different procedures to be carried out. The US self-acquisitions in Position 1 and Position 3 were effectively carried out by the subjects in a short time (~10 sec). Indeed, for these positions, even non-skilled operators could easily recognize the anterior and posterior cartilage features. To perform the self-acquisition in Position 3 with the leg completely extended, the subject found the sitting position more comfortable than standing. Differently, the acquisitions in Position 2 resulted rather hard to manage by non-expert operators because the anatomical structures of interest are more difficult to be interpreted. Such difficulty resulted in a higher acquisition time (~20 sec) needed to acquire clinical effective US images in Position 2. Moreover, the exact positions of the openings dedicated to imaging such structures, especially scan 2B, were subjected to the highest variability between the different subjects, as shown by the probe location measurements in Table 1. It is worth mentioning that Doppler analysis could be also informative in this position for the Hoffa’s adipose body assessment and also for synovial inflammation detection, by employing US probes with this function enabled. In a future perspective, a caregiver could help the patient during the acquisition of US images, thus reducing the acquisition times for imaging in Position 2 and the discomfort for the patient in Position 3, for which the visual feedback is hampered.

Overall, the B-mode images taken by untrained subjects wearing the wearable brace (Figure 4-c) are comparable to the B-mode images taken by an expert clinician shown in Figure 1-c, demonstrating the possibility to analyze through US imaging the knee in a stable and repeatable way.

Thus, the proposed wearable brace proved to be useable as a guidance system intended for US image acquisition also by untrained operators, paving the way for the use of tele-ultrasonography in OA diagnosis.

Although research efforts regarding portable US are quite limited, some interesting results demonstrated the potential of tele-ultrasonography to positively influence the management and treatment in some clinical fields (*e.g.,* obstetrics and gynaecology) [8]. In tele-ultrasonography, US images and videos are stored by a trained operator and forwarded to a specialized clinician for interpretation. Alternatively, US images can be acquired in real-time by an untrained user under the remote supervision of an expert.

In a future perspective of a remote OA diagnosis, a clinician will acquire reference US images during a first medical examination. After this first evaluation, the follow-up is expected to be performed remotely: the patient (or a caregiver that helps the patient) may acquire US images by using the brace, guided by the reference images previously acquired by the clinician. The collected images will be then forwarded to a specialized clinician and evaluated for the final diagnosis. Therefore, the proposed procedure is not meant to substitute the role of the clinician: the experience of the clinician is always needed to evaluate the results and make the diagnosis. On the other hand, tele-ultrasonography may speed-up the process of diagnosis, allowing more frequent follow-up controls, and providing a non-invasive, fast and repeatable examination of cartilage degeneration and joint inflammation over time, thus improving the quality of patient care.

However, the performance of tele-ultrasonography is strictly dependent on the quality of the collected US images: the use of standardized acquisition protocols could significantly help users to obtain accurate and informative US images [8], [13]. In this regard, our results represent a preliminary but interesting first step toward the use of a tele-ultrasound platform for the diagnosis of OA.

The correct setting of the acoustic parameters is an important factor, which may affect the quality of US images. For all the positions the acoustic parameters (such as depth, frequency, Time Gain Compensation) were preliminary set based on the know-how of expert clinicians. In this study, the same settings were suitable for the three subjects. However, in the future clinical scenario, these parameters could be personalized for each patient in the first examination. Then, each patient would use his/her own pre-setting during the remote follow-up.

The correct positioning of the subject is also a crucial aspect, which can influence the acquisition of clinically informative images. In this study, precise guidelines were given by expert clinicians in the field and a goniometer was used to ensure the correct positioning. In the future clinical scenario, alternative solutions could be employed: for example, a dedicated adjustable bench with an integrated goniometer could help the subject to reach and maintain the correct positions in a repeatable way.

The authors are aware that further efforts need to be faced to make such a technique effective in the routine clinical practice for OA diagnosis. Future improvements may include the use of embedded sensors to further standardize the probe handling procedures during the acquisition: for example, force sensors may help the control of the pressure exerted on the skin [14], while an inclinometer sensor [15] may regulate the angle of the probe with respect to the knee. In this study, all the US images were acquired by placing the probe at 90° with respect to the skin (based on clinician’s indications). The brace inserts shape allowed keeping fixed such inclination. An indication on how the quality of the US images can be affected by varying the probe inclination, is given in Figure S9 of the supplementary material, demonstrating that 90° is a suitable angle for this analysis. Moreover, automatic algorithms could be implemented to precisely identify among the scans performed by the patients the one most similar to the scan performed by the clinician.

In this study, three subjects with a similar height were analyzed. To assess the usability of the proposed wearable brace on subjects with different heights, two additional subjects were recruited: one much taller (subject 4, height: 190 cm) and one much shorter (subject 5, height: 165 cm) with respect to the average height considered in this study (181.3 cm). The preliminary results, shown in the supplementary material (Figure S10 and Figure S11), suggest that the dimensions and positions of the brace opening are suitable also for such different subject heights. Future efforts may be focused toward making the brace more comfortable and adaptable to subjects of different size, in terms of wearability. For example, the use of an elastic Velcro may allow regulating the diameter of the brace to different knee girths. Future trials will be needed to confirm these results and to further assess the feasibility of such a technique on a broader range of subjects, also including OA patients. Moreover, this approach may be extended in the future to the assessment of other degenerative and inflammatory pathologies, *e.g.* rheumatoid arthritis.

## V. Conclusion

In this study, we proposed a wearable tool and a standard protocol for the US examinations of knee injuries. Starting from clinical specifications a wearable brace was developed to guide the correct placements of the US probe to obtain a precise evaluation of the cartilage and other structures of interest. Overall, three positions were recognized to achieve a global assessment of the knee, with particular regards on OA changes and joint inflammation. The feasibility of the proposed wearable brace and the standardized acquisition protocol was tested on three healthy subjects, which were asked to acquire US images similar to the reference images provided by the clinician. The untrained operators were able to acquire informative and correct images in the Position 1, 2 and 3. However, the acquisitions in Position 2 appeared more difficult to manage and thus the subjects required more time to achieve the correct images. Future efforts will focus on the improvement of such standardization method, with the final aim to validate such technology on a wider range of patients. Our results may also open up to the use of tele-ultrasonography, allowing a repeatable, non-invasive, low-cost and fast assessments of the knee joint.

## Informed Consent

Authors declare as follows.

• The study has been carried out in compliance with the current applicable ethical-legal framework (EU Charter of Fundamental Rights, Declaration of Helsinki, EU Reg. 2016/679 General Data Protection Regulation “GDPR”). Ethics committee approval is not required as it is an observational study.
• Participation of volunteers was conditioned to the collection of freely-given informed consent. The information sheet included transparent and exhaustive information regarding the project, partners involved in the observational study, benefits, possible risks, incidental findings procedure, insurance details, information privacy under article 13 GDPR. Only adults and healthy participants have been recruited.
• The collected personal data have been pseudonymized and stored in a limited accessed folder. Any re-use of those personal data will be possible only under the conditions stated in articles 5 and 89 GDPR. Authors are committed to specific confidential obligations, according to the “Ethics rules on personal data processing for research and statistics purposes” issued by the Italian Data Protection Authority.

## Supplementary Materials

Supplementary Materials
