# Supplementary material for: Design, Development and Validation of a Knee Brace to Standardize the US Imaging Evaluation of Knee Osteoarthritis
Source: IEEE J Transl Eng Health Med. 2021 Dec 22;10:1800308. doi: 10.1109/JTEHM.2021.3137628 (PMC8979410; doi:10.1109/JTEHM.2021.3137628)
Supplement: Supplementary Materials [file supp1-3137628.pdf]

SUPPLEMENTARY MATERIAL

Design, development and validation of a knee brace to standardize the US imaging evaluation of knee osteoarthritis

A. Sorriento[1,2], A. Cafarelli[1,2], P. Spinnato[3], A. Russo[3], G. Lisignoli[3], F. Rabusseau[4], P. Cabras[4], E. Dumont[4], L. Ricotti[1,2]*

[1]The BioRobotics Institute, Scuola Superiore Sant'Anna, 56127 Pisa, Italy

[2]Department of Excellence in Robotics & AI, Scuola Superiore Sant'Anna, 56127 Pisa, Italy

[3]IRCCS Istituto Ortopedico Rizzoli, Diagnostic and Interventional Radiology, Bologna, Italy

[4]IGT, Image Guided Therapy, Pessac, France

*Corresponding author

Angela Sorriento

The BioRobotics Institute, Scuola Superiore Sant'Anna

Viale R. Piaggio 34, 56025, Pontedera (PI), Italy

e-mail: angela.sorriento@santannapisa.it

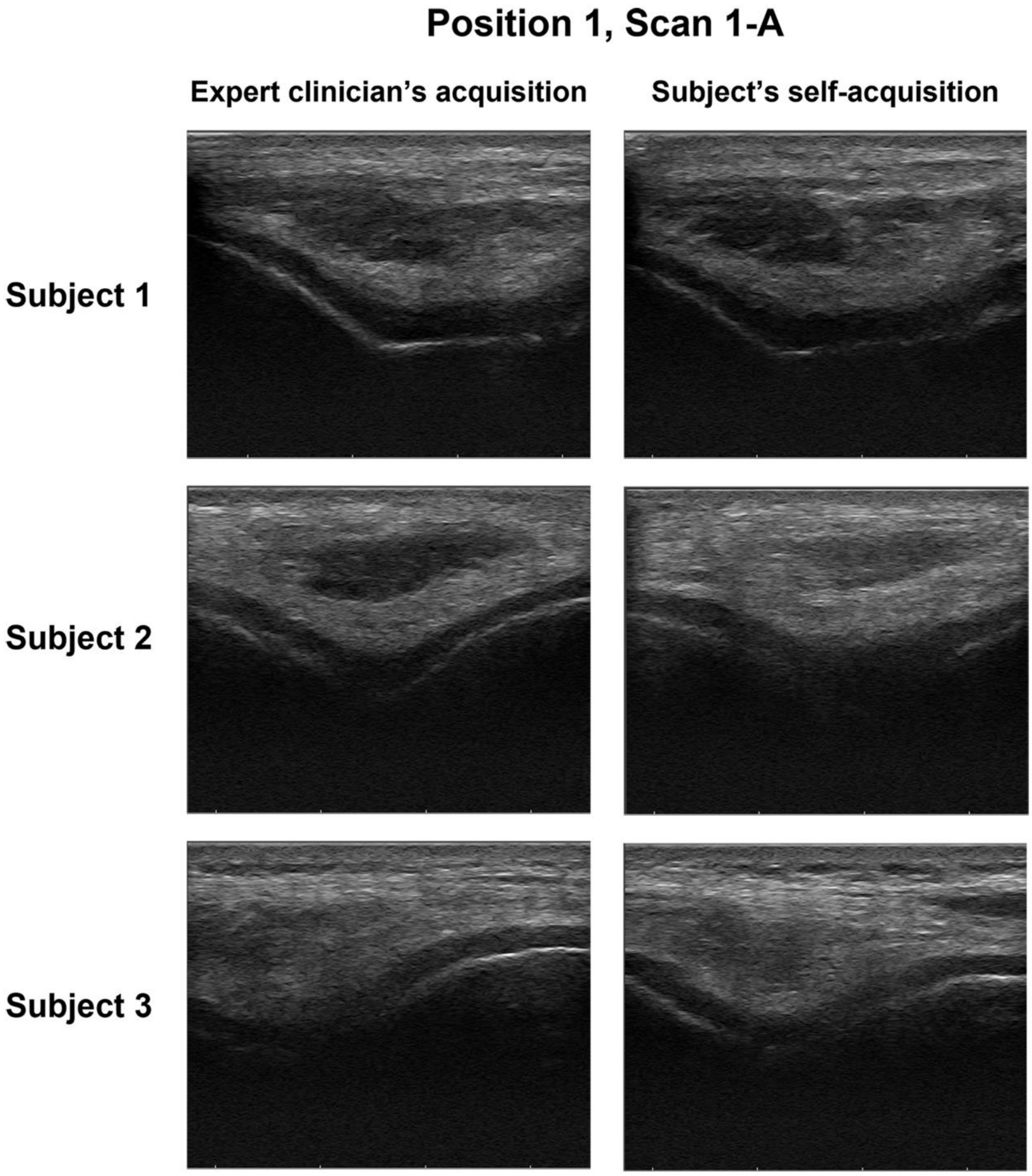

Figure S 1 – Comparison of US images in position 1, scan 1-A. *In this figure a direct comparison between the reference US images acquired by the clinician (left panel) and the US images acquired by the subjects (right panel) while wearing the knee brace is presented for the position 1, scan 1-A.*

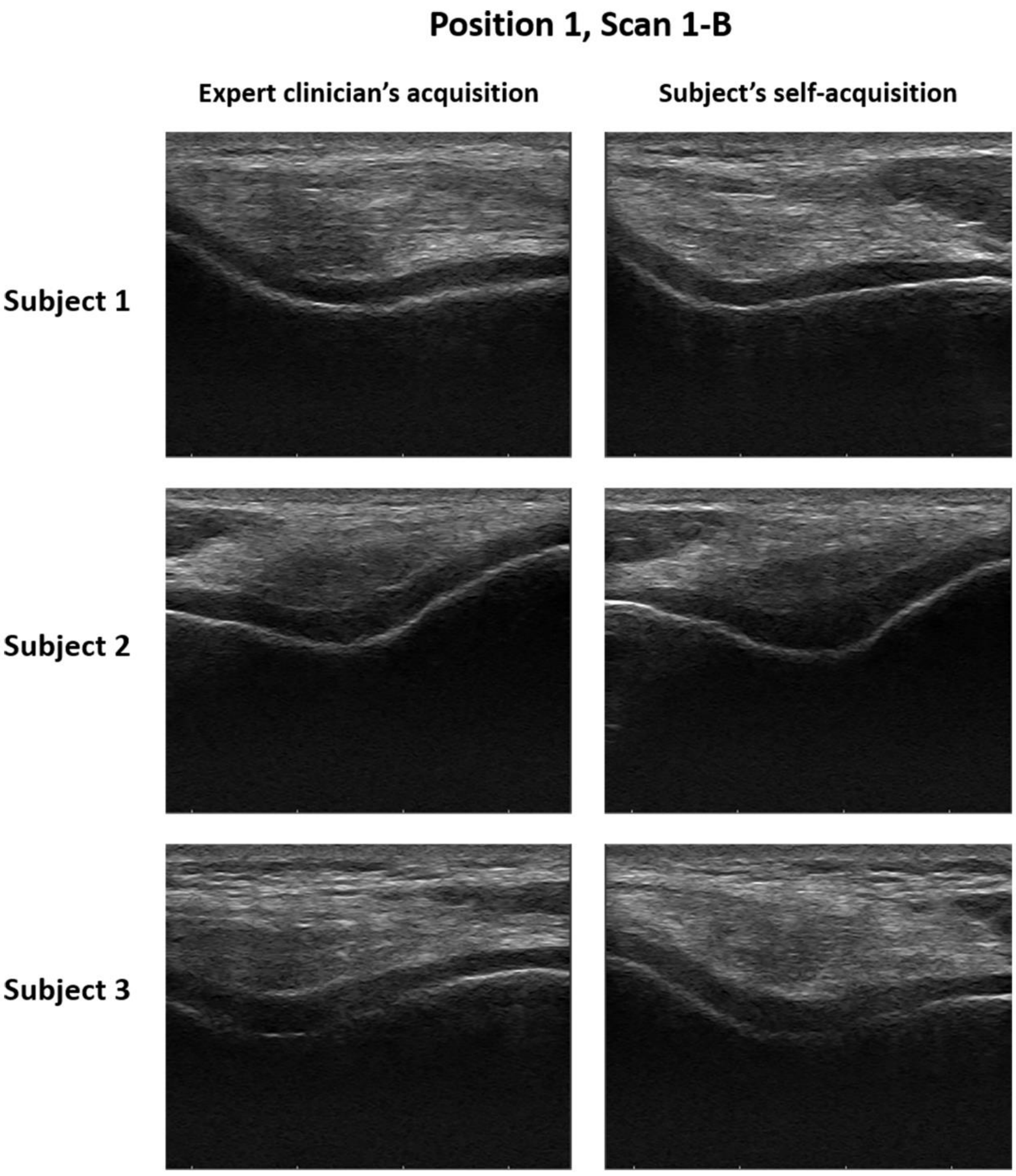

Figure S 2 - Comparison of US images in position 1, scan 1-B. *In this figure a direct comparison between the reference US images acquired by the clinician (left panel) and the US images acquired by the subjects (right panel) while wearing the knee brace is presented for the position 1, scan 1-B.*

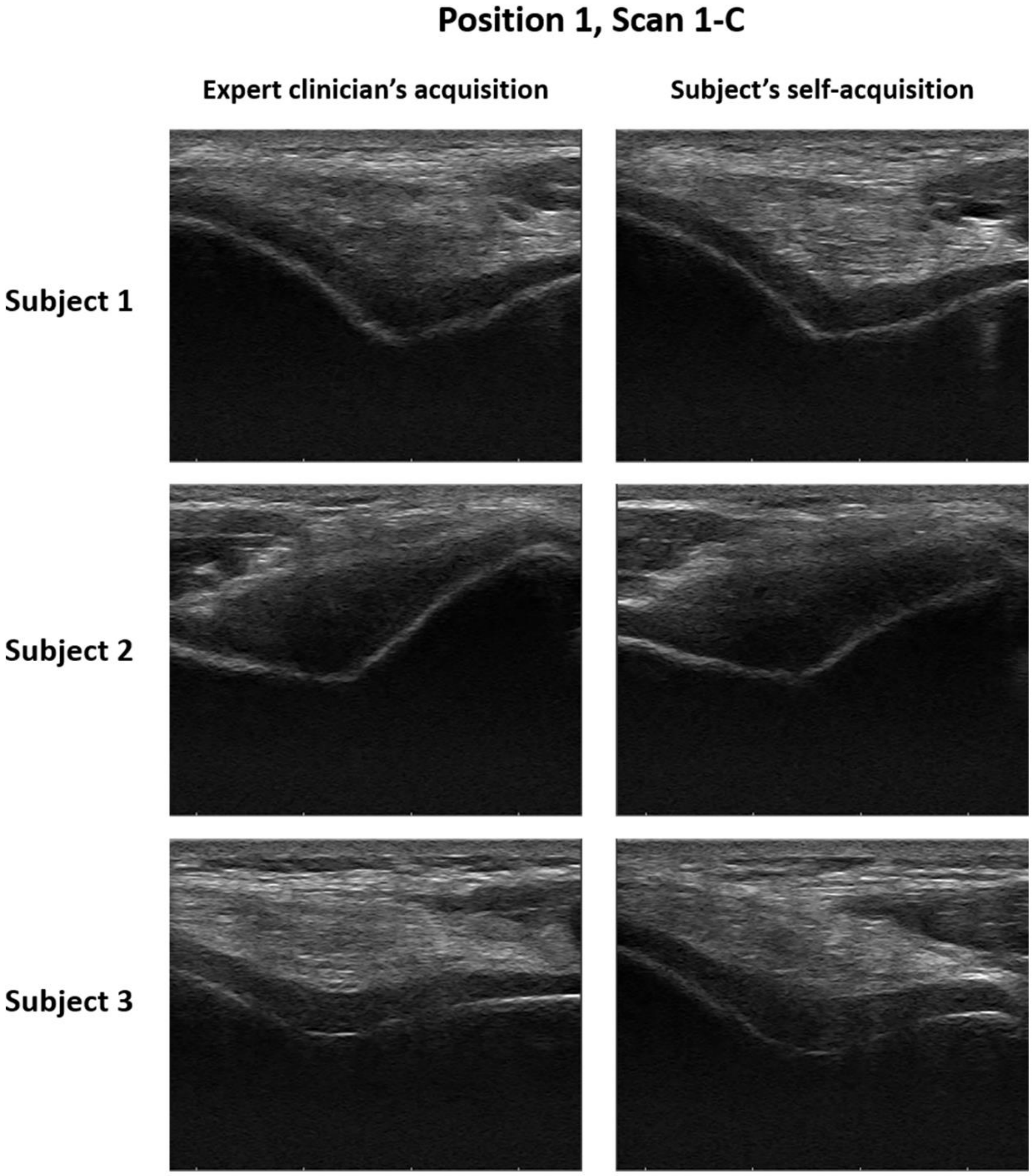

Figure S 3 - Comparison of US images in position 1, scan 1-C. *In this figure a direct comparison between the reference US images acquired by the clinician (left panel) and the US images acquired by the subjects (right panel) while wearing the knee brace is presented for the position 1, scan 1-C.*

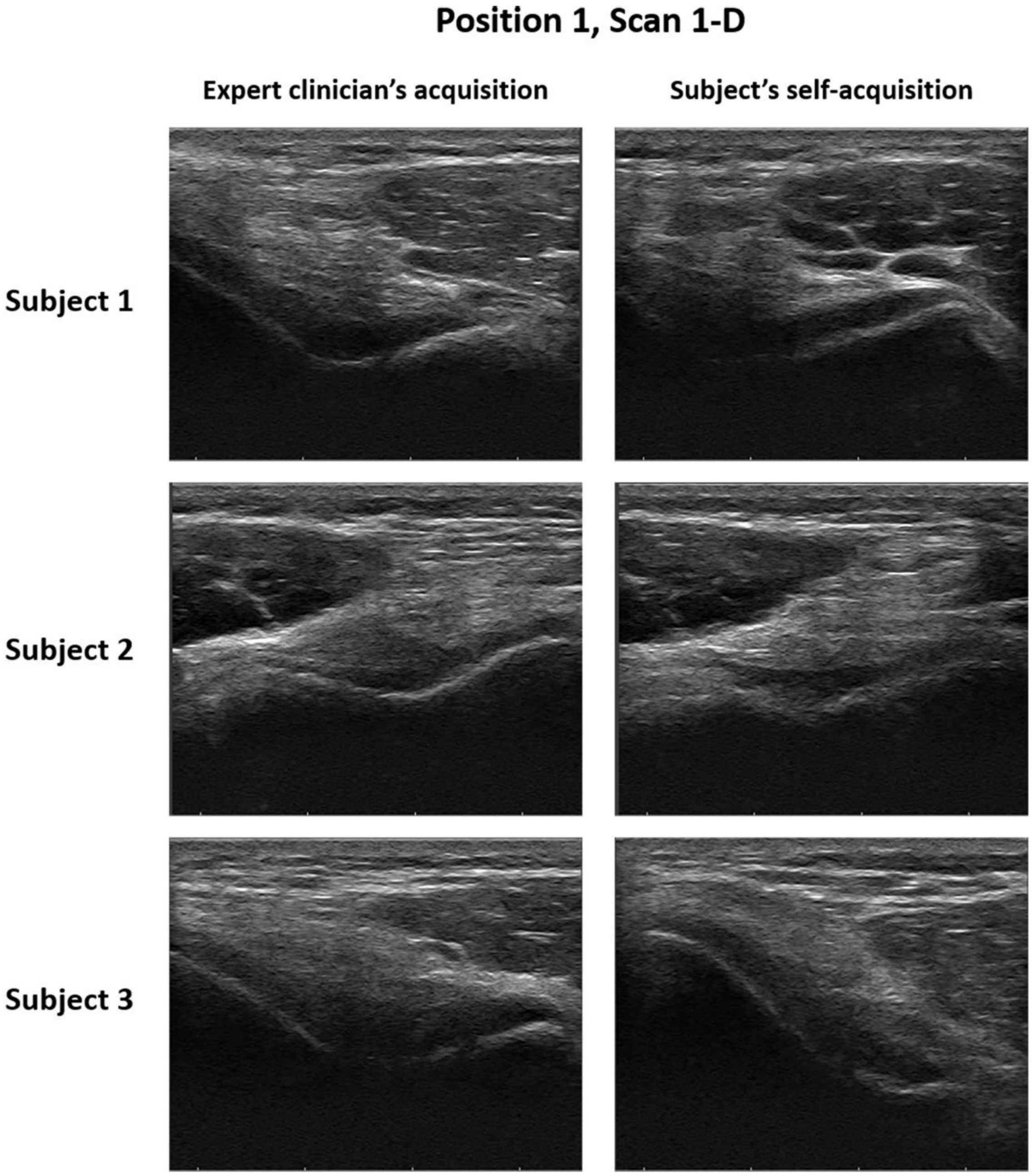

Figure S 4 - Comparison of US images in position 1, scan 1-D. *In this figure a direct comparison between the reference US images acquired by the clinician (left panel) and the US images acquired by the subjects (right panel) while wearing the knee brace is presented for the position 1, scan 1-D.*

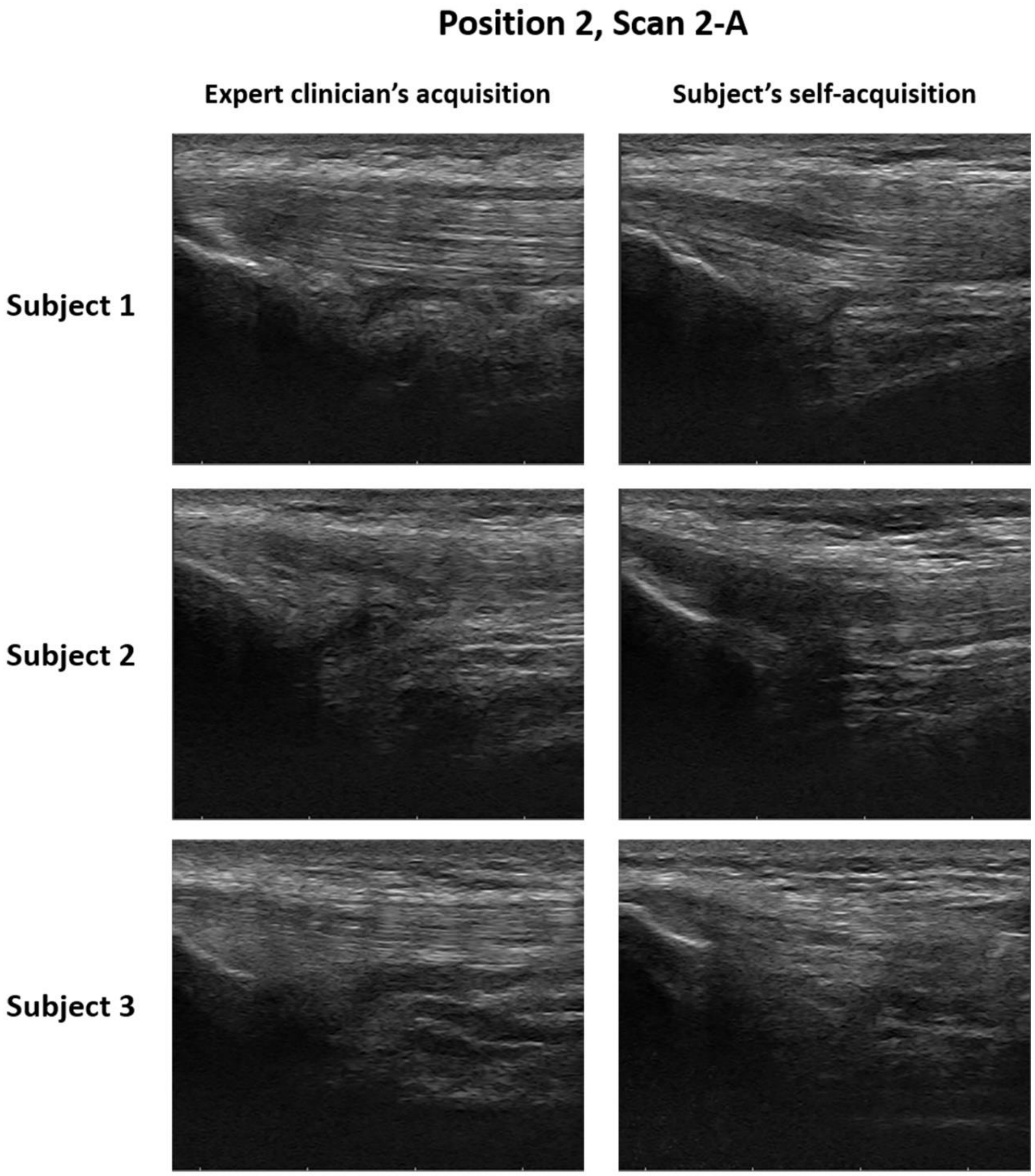

Figure S 5 - Comparison of US images in position 2, scan 2-A. *In this figure a direct comparison between the reference US images acquired by the clinician (left panel) and the US images acquired by the subjects (right panel) while wearing the knee brace is presented for the position 2, scan 2-A.*

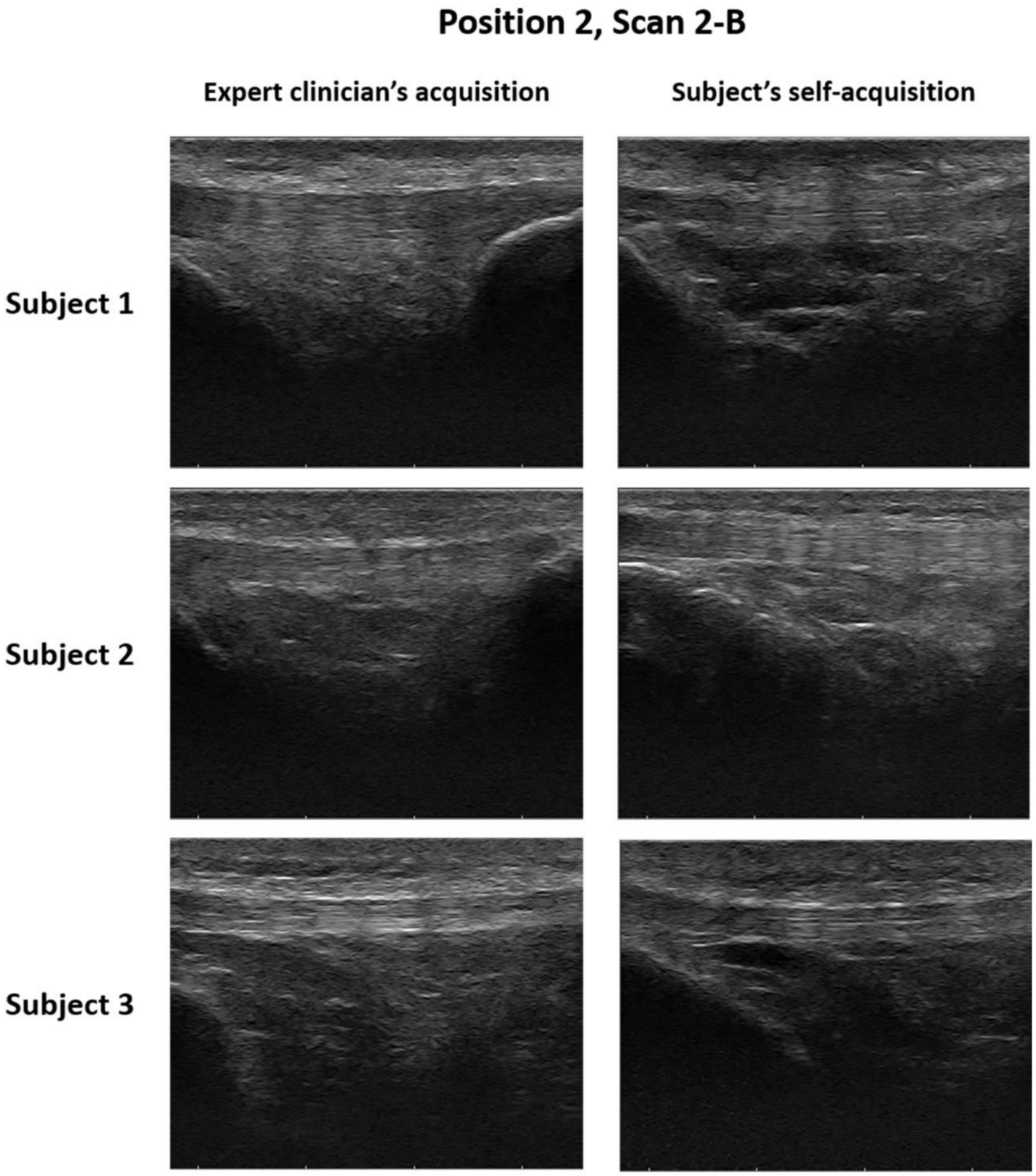

Figure S 6 - Comparison of US images in position 2, scan 2-B. *In this figure a direct comparison between the reference US images acquired by the clinician (left panel) and the US images acquired by the subjects (right panel) while wearing the knee brace is presented for the position 2, scan 2-B.*

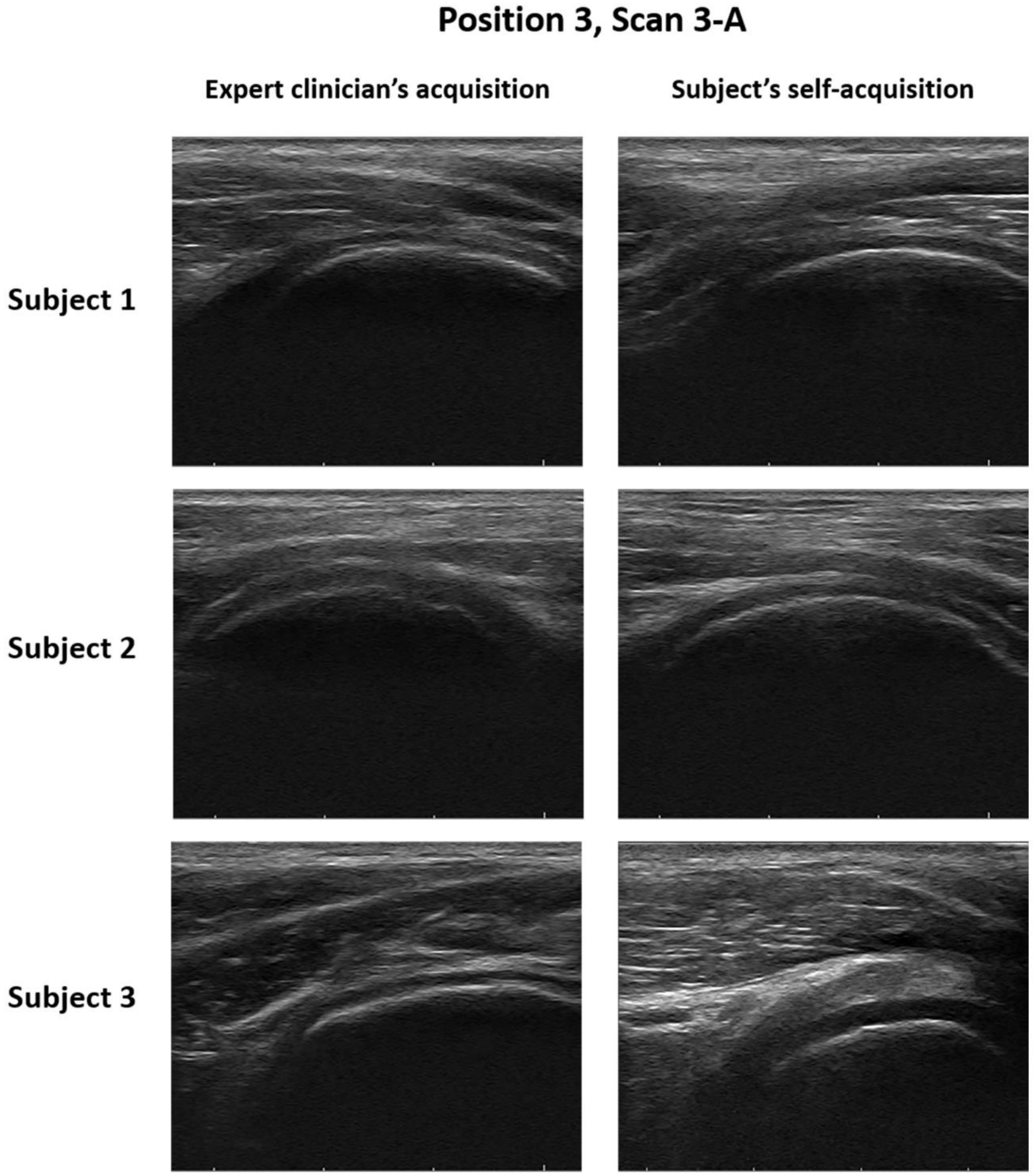

Figure S 7 - Comparison of US images in position 3, scan 3-A. *In this figure a direct comparison between the reference US images acquired by the clinician (left panel) and the US images acquired by the subjects (right panel) while wearing the knee brace is presented for the position 3, scan 3-A.*

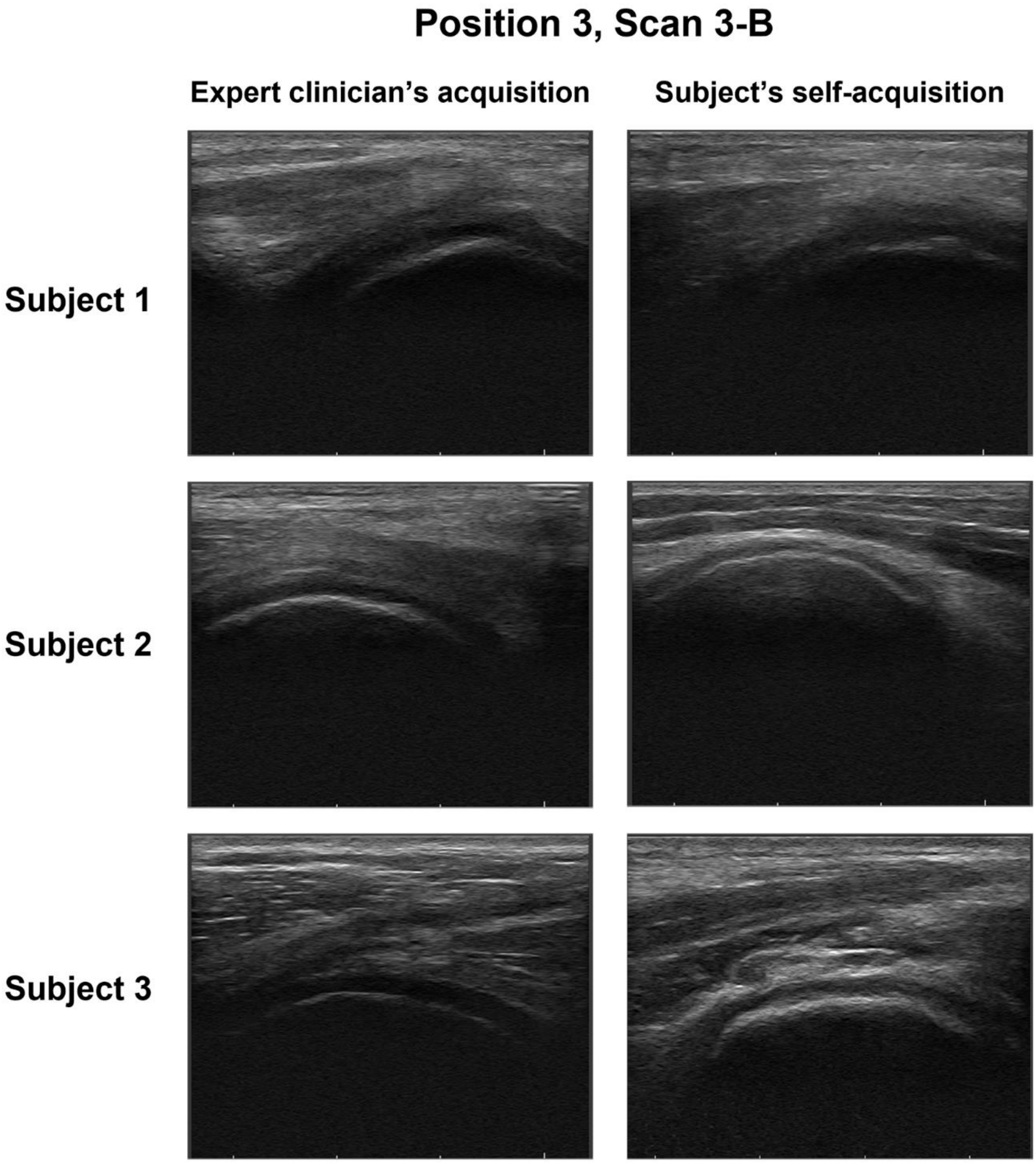

Figure S 8 - Comparison of US images in position 3, scan 3-B *In this figure a direct comparison between the reference US images acquired by the clinician (left panel) and the US images acquired by the subjects (right panel) while wearing the knee brace is presented for the position 3, scan 3-B.*

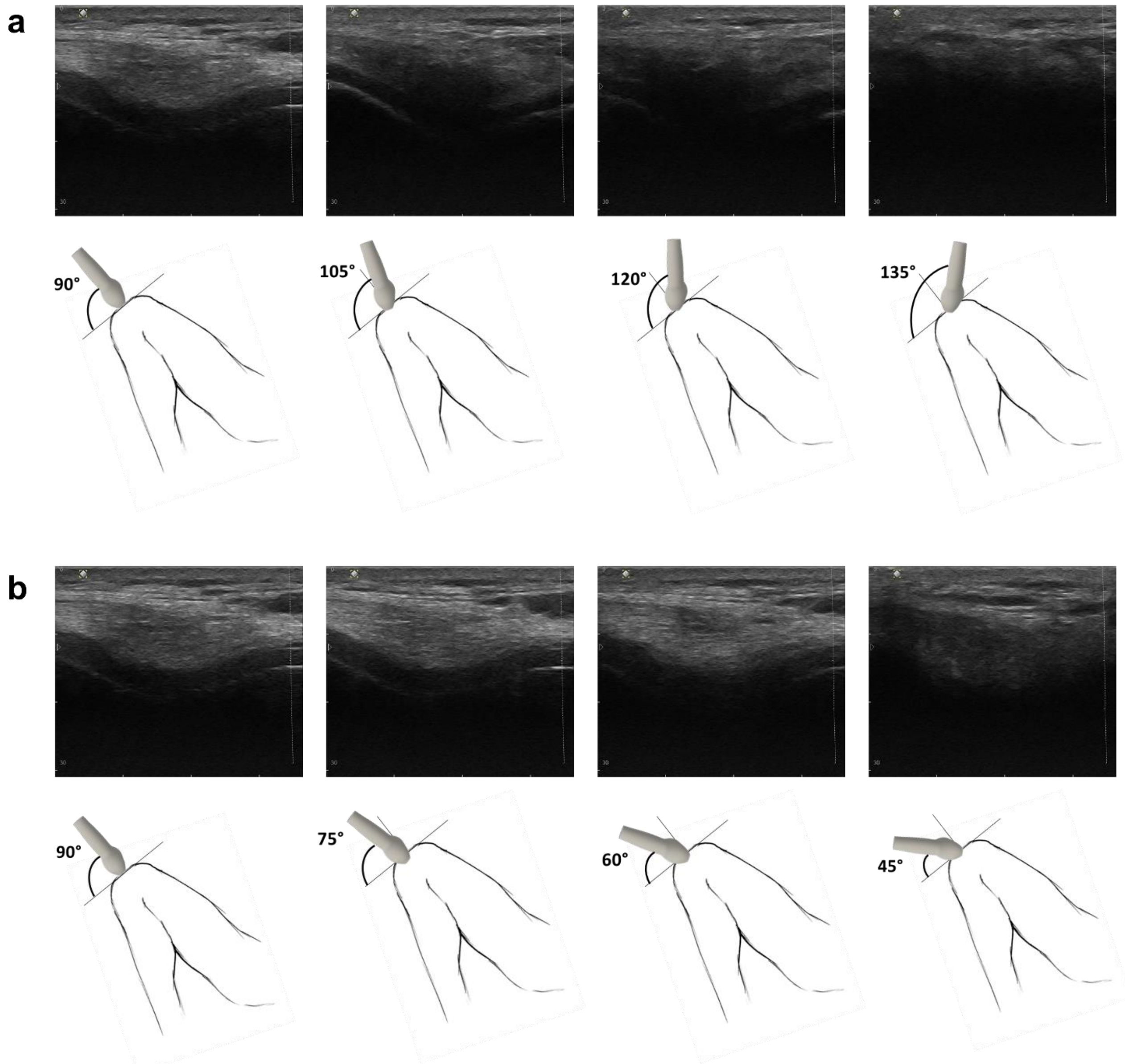

Figure S 9 - Ultrasound images acquired in Pos1 – scan 1A on one subject, at different inclinations of the probe. *Starting from the perpendicular position (90°), the probe was tilted in both positive (a) and negative (b) directions with a step size of 15°. At each angle, a US image was acquired by the subject and compared to the one obtained keeping a perpendicular position. When the probe tilt is low (± 15) the cartilage structures can still be recognized. However, when the probe inclination increases (± 30°) the loss of cartilage information increases, until a complete loss of this information for high inclinations (± 45°).*

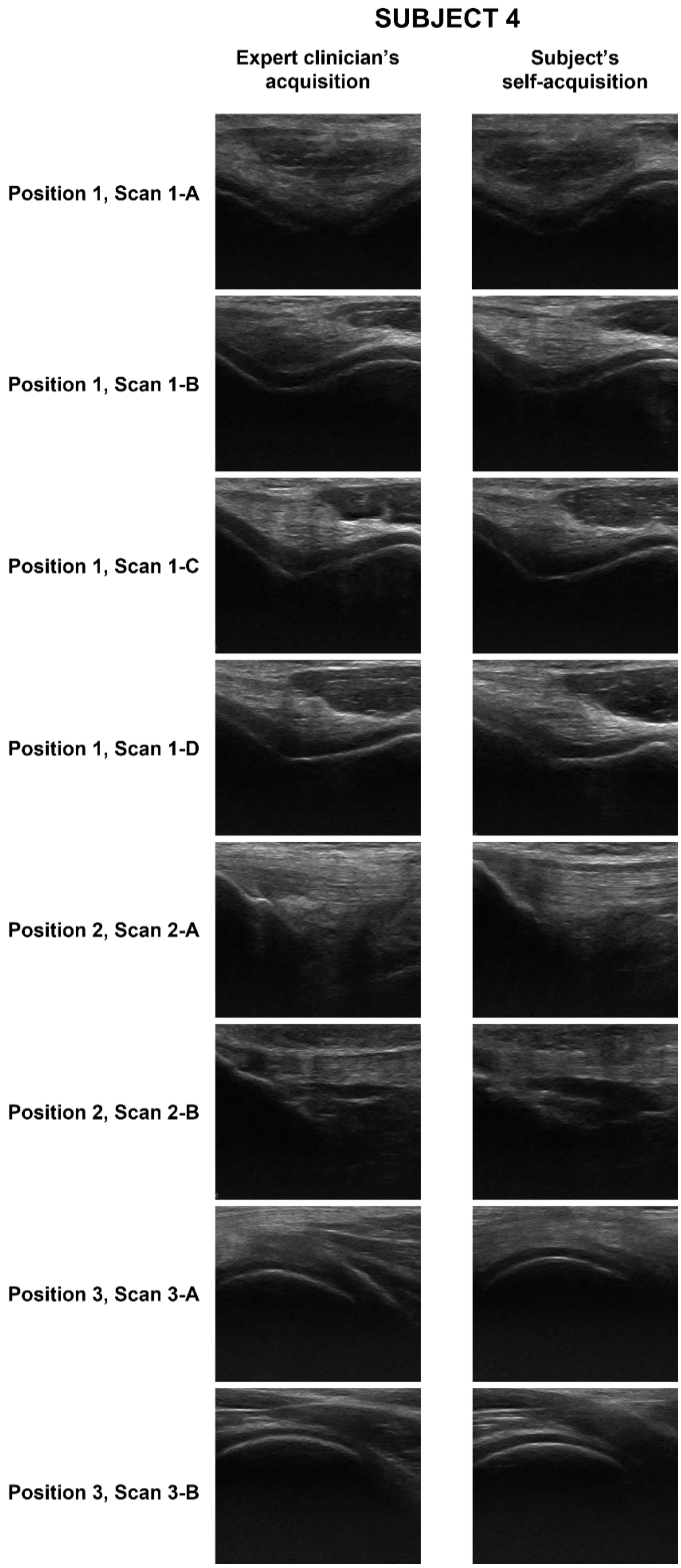

Figure S 10 – Comparison between US images taken by clinicians and taken by the subject in all probe locations, for a tall subject (height: 190 cm, weight: 77 kg). *A direct comparison between the reference US images acquired by the clinician (left) and the ones acquired by the subject while wearing the knee brace (right) is reported for all the target positions. These preliminary results suggest that the dimensions and positions of the brace openings are suitable also for such subject height.*

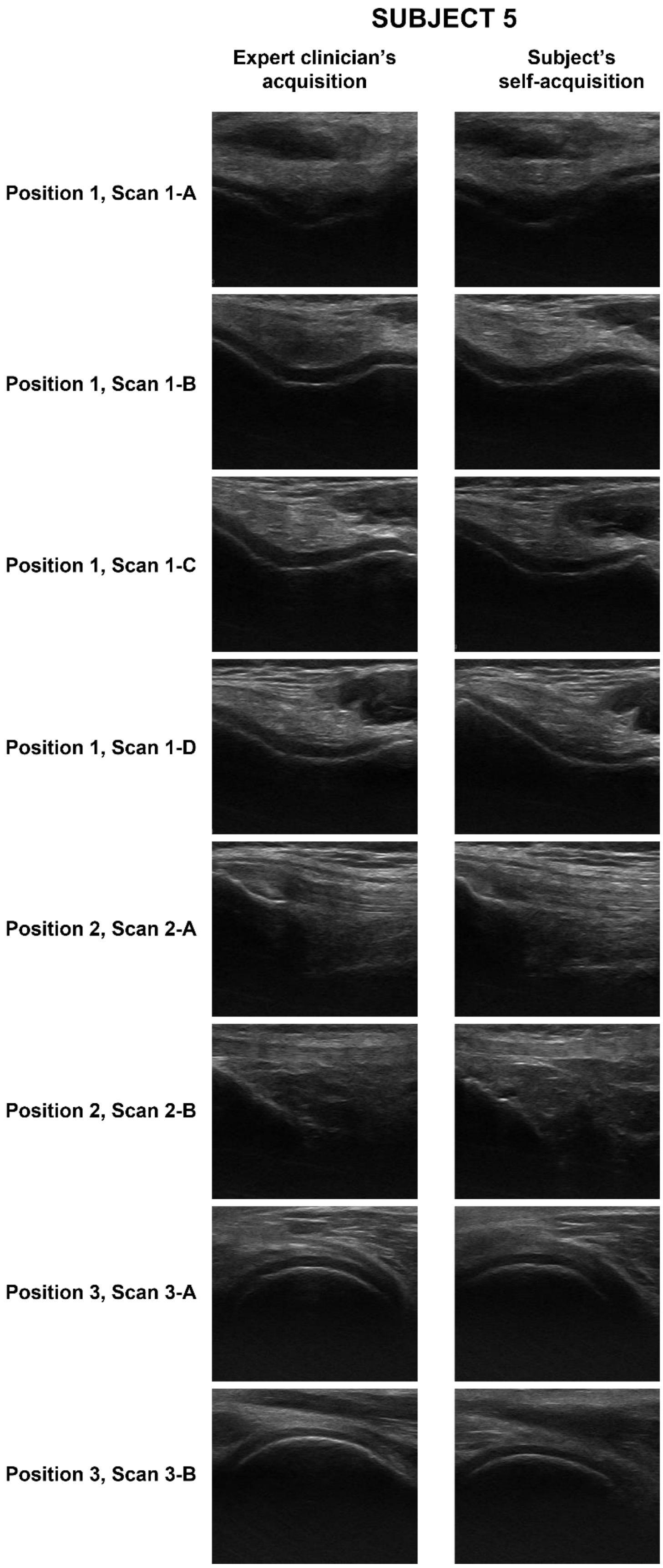

Figure S 11 - Comparison between US images taken by clinicians and taken by the subject in all the positions for a short subject (height: 165 cm, weight: 60 kg). *A direct comparison between the reference US images acquired by the clinician (left) and the ones acquired by the subject while wearing the knee brace (right) is reported for all the target positions. These preliminary results suggest that the dimensions and positions of the brace openings are suitable also for such subject height.*
